# Resolving the spatial organization of fetal liver hematopoiesis by SeekSpace

**DOI:** 10.1186/s13619-025-00234-0

**Published:** 2025-04-22

**Authors:** Xinyu Thomas Tang, Lin Veronica Chen, Bo O. Zhou

**Affiliations:** 1 Key Laboratory of Multi-Cell Systems, Shanghai Institute of Biochemistry and Cell Biology, Center for Excellence in Molecular Cell Science, Chinese Academy of Sciences, University of Chinese Academy of Sciences, Shanghai, 200031 China; 2 State Key Laboratory of Experimental Hematology, Institute of Hematology & Blood Diseases Hospital, Chinese Academy of Medical Sciences, Tianjin, 300020 China

**Keywords:** Hematopoietic stem and progenitor cells, Fetal liver, Hematopoiesis, Spatial transcriptomic, Niche

## Abstract

The fetal liver is the primary site for the expansion of hematopoietic stem and progenitor cells (HSPCs) during fetal hematopoiesis. However, the spatial organization of different hematopoietic progenitor populations within the fetal liver remains poorly characterized. In this study, we utilized SeekSpace, a high-resolution single-nucleus spatial transcriptomics platform, to map the spatial distribution of hematopoietic stem cells and multipotent progenitor cells (HSC/MPPs) and downstream restricted progenitors (RPs) in relation to other hematopoietic and stromal cell populations in the fetal liver at embryonic day 13.5. Using SeekSpace, we constructed a detailed single-cell spatial transcriptomic atlas of fetal liver hematopoiesis, revealing that both HSC/MPPs and many RPs undergo active expansion in the fetal liver, a process distinct from their behavior in adult bone marrow. Proximity analysis and in situ imaging demonstrated that HSC/MPPs expansion occurs in close association with macrophages and endothelial cells throughout the fetal liver, supported by signaling pathways involving IGF and collagen. In contrast, RPs exhibited no specific spatial proximity to other cell populations during their expansion. Collectively, this study provides a comprehensive resource for understanding the spatial and molecular mechanisms underlying HSC/MPPs and RP expansion during fetal liver hematopoiesis.

**Supplementary Information:**

The online version contains supplementary material available at 10.1186/s13619-025-00234-0.

## Background

Hematopoietic stem and progenitor cells (HSPCs) are essential for the lifelong maintenance of hematopoiesis through continuous production of mature blood cells (Laurenti and Gottgens 2018; Patel et al. 2022). During embryonic development, HSPCs arise from a specialized subset of endothelial cells (ECs) through a process known as endothelial-to-hematopoietic transition (EHT) (Chen et al. 2009; Medvinsky and Dzierzak 1996). In mice, this process occurs between embryonic days (E) 9.5 and 11.5, generating hundreds of hematopoietic cluster cells (HCCs) (Ganuza et al. 2017). These HCCs subsequently migrate to the fetal liver, where they proliferate and establish a robust HSPC pool that sustains hematopoiesis throughout life (Ema and Nakauchi 2000; Morrison et al. 1995). During this brief embryonic phase, HSPCs are required to rapidly expand to support fetal hematopoiesis (Patel et al. 2022; Yokomizo et al. 2022; Zhang et al. 2021b). However, the organization of HSPC expansion within the fetal liver remains poorly understood.

The spatial organization of HSPCs, known as niche, is fundamental to the regulation of their function and behavior. In adults, HSPCs are maintained within a perivascular niche in the bone marrow, which is composed of a diverse array of cellular components (Morrison and Scadden 2014; Wei and Frenette 2018). During development, the fetal liver acts as the primary site of hematopoiesis, facilitating both the maturation and expansion of HSPCs (Gao and Liu 2018). Recent studies have identified several candidate cell populations within the fetal liver that may serve as niches for HSPCs, including Nestin^+^NG2^+^ periportal stromal cells, hepatic stellate cells, endothelial cells, and macrophages (Gao et al. 2022; Kayvanjoo et al. 2024; Khan et al. 2016; Lee et al. 2021). Recent study has demonstrated that hematopoietic stem and progenitors have independent origins before migrating into the fetal liver (Yokomizo et al. 2022), which raises a new question of whether they have similar niche requirements for expansion in the fetal liver.

Advances in spatial transcriptomics (ST) have enabled the high-resolution analysis of in situ gene expression within intact tissues (Chen et al. 2022; Chen et al. 2015; Liu et al. 2020; Rodriques et al. 2019; Stahl et al. 2016). A recent study utilized spatial transcriptomics to identify the expansion units of hematopoietic stem cells and multipotent progenitor cells (HSC/MPPs) in the fetal liver (Gao et al. 2022). However, traditional spatial transcriptomics methods, such as 10 × Visium, rely on large capture spots (tens to hundreds of μm in diameter) that often encompass multiple cells, leading to mixed RNA signals and ambiguity in cell-type identification. In this study, we employed a spatial barcode-based single nucleus ST, named SeekSpace, to construct a comprehensive spatial transcriptomic atlas of fetal liver hematopoiesis. SeekSpace achieves true single-cell resolution with its ultra-high-density spatial chip (900 nm label clusters), eliminating RNA cross-contamination and enabling precise gene expression localization without algorithmic imputation. SeekSpace revealed the active expansion of HSPCs distinct from that observed in adult bone marrow hematopoiesis. Furthermore, in situ imaging of hematopoietic stem cells (HSCs) uncovered a dramatic expansion of HSCs within a niche defined by macrophages and endothelial cells at E13.5.

## Results

### Single-nucleus spatial transcriptomic atlas of fetal liver

**Fig. 1:**
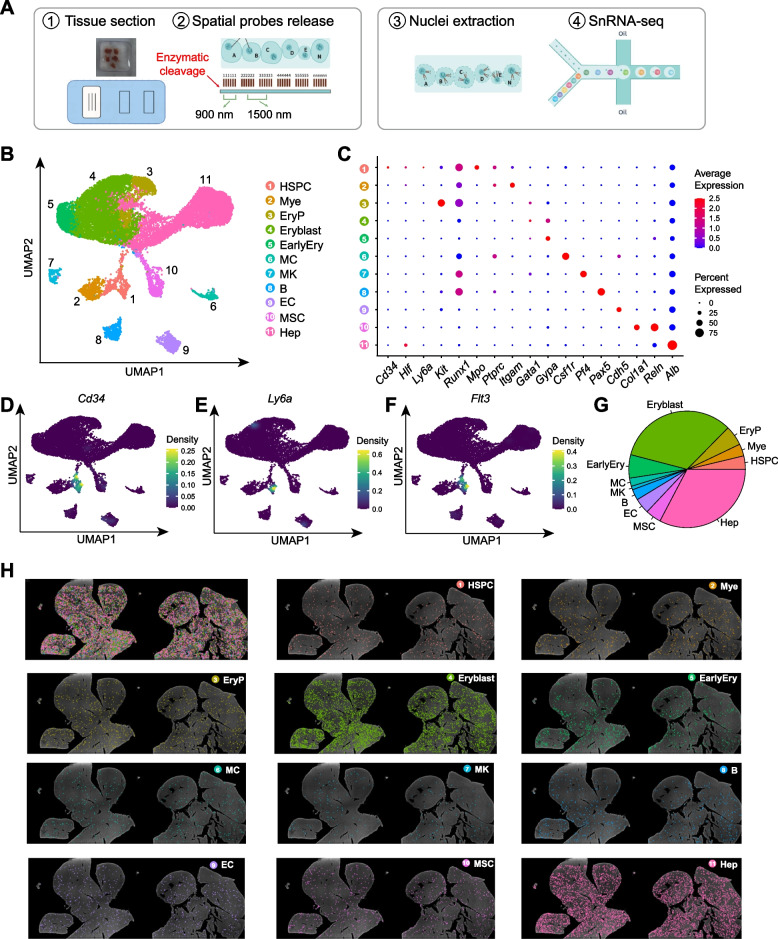
Spatial transcriptomic landscape of fetal liver. **A** Schematic illustrating the workflow for single-cell spatial transcriptomics. **B** Uniform manifold approximation and projection (UMAP) visualization of unsupervised clustering of nuclei isolated from an E13.5 fetal liver section. **C** Dot plot showing the expression levels of representative marker genes across different clusters. **D**-**F** Nebulosa density plots showing the expression levels of *Cd34* (**D**), *Ly6a* (**E**) and *Flt3* (**F**). **G** Pie chart showing the proportion of different clusters. **H** Spatial distribution of various clusters within the E13.5 fetal liver section

To better understand the spatial organization of fetal hematopoiesis and its associated niches, we employed spatial barcode-based single-cell ST (SeekSpace) to generate a comprehensive spatial transcriptomic atlas of the E13.5 fetal liver. SeekSpace was developed on the basis of the Slide-tag ST method (Russell et al. 2024). SeekSpace offers higher spatial resolution than Slide-seq by using 900 nm tag clusters instead of 10 µm beads, enabling more precise cellular localization. Additionally, its enzymatic tag release method preserves RNA quality better than Slide-seq’s UV-based release, which can degrade RNA (Fig. 1A). After tissue processing, cleaved spatial barcode fragments containing polyA sequences were incorporated into the nuclei and subsequently underwent microdroplet-based single-nucleus RNA sequencing (snRNA-seq), enabling the simultaneous capture of both the nuclear transcriptome and spatial information.

We obtained a total of 24,343 nuclei from two fetal liver samples on the same slide, of which 17,984 passed the quality control, with an average of 578 genes and 2320 unique molecular identifiers (UMIs) per nucleus (Fig. S1). Then, uniform manifold approximation and projection (UMAP) was performed to investigate cellular heterogeneity, and 11 distinct clusters were identified by unbiased clustering (Fig. 1B-F). Notably, the hematopoietic clusters included HSPCs (expressing *Cd34*, *Hlf*, *Ly6a*, *Kit*, *Runx1* and *Mpo*), myeloid cells (Mye, expressing *Ptprc* and *Itgam*), erythroid progenitors (EryP, expressing *Kit* and *Gata1*), erythroblasts (Eryblast, expressing *Gata1* and *Gypa*), early erythrocytes (EarlyEry, expressing *Gypa*), macrophages (MC, expressing *Csf1r*), megakaryocytes (MK, expressing *Pf4*) and B cells (B, expressing *Pax5*). Non-hematopoietic clusters included endothelial cells (EC, expressing *Cdh5*), mesenchymal stromal cells (MSC, expressing *Col1a1 and Reln*) and hepatoblasts (Hep, expressing *Alb*). Erythropoiesis was particularly active within the fetal liver, leading to the enrichment of erythrocyte and hepatoblast populations (Fig. 1G).

To explore the spatial distribution of these distinct cell populations, we mapped the 11 clusters to the nuclear imaging data according to the spatial barcodes of individual nucleus (Fig. 1H). Most cell populations exhibited a dispersed distribution throughout the liver sections, except the Eryblast cluster, which showed notable aggregation at specific foci (Fig. 1H). Taken together, our single-cell spatiotemporal transcriptomic profiling provides a comprehensive spatial organization atlas of fetal liver hematopoiesis.

### Single-nucleus spatial transcriptomic profiling of fetal liver HSPCs

**Fig. 2:**
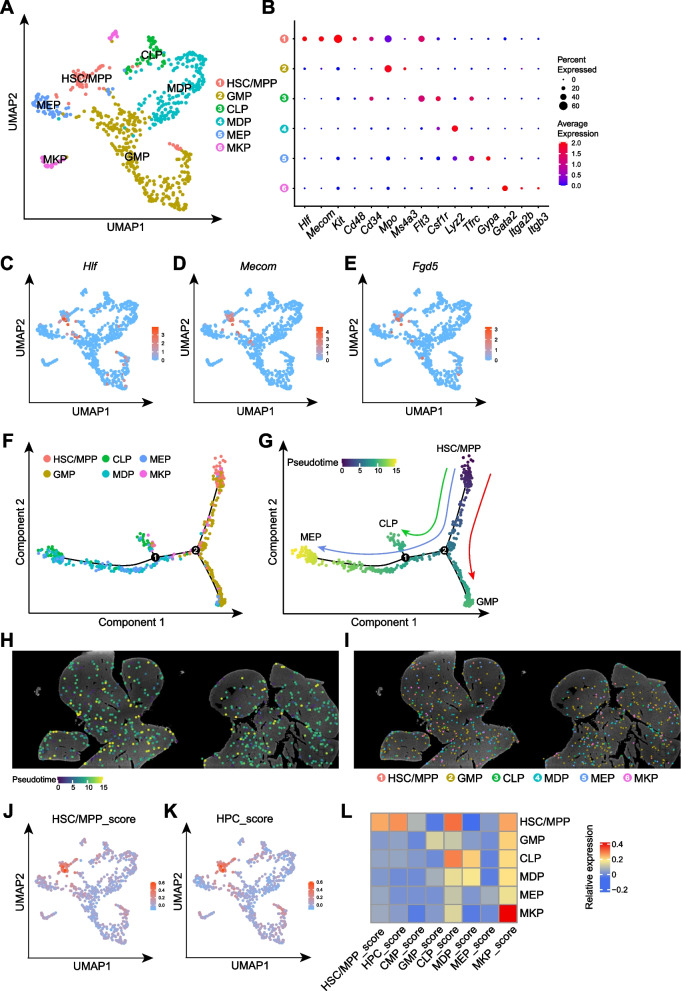
Identification of distinct HSPC subtypes in fetal hematopoiesis. **A** UMAP visualization of unsupervised clustering of hematopoietic stem and progenitor cells (HSPCs) isolated from E13.5 fetal liver section. **B** Dot plot showing the expression levels of representative marker genes in different HSPCs clusters. **C**-**E** UMAP plots displaying the expression of representative genes in different HSPCs clusters. **F** and **G** Pseudotemporal ordering of the HSPC subpopulations inferred by Monocle2, with clusters (**F**) and pseudotime (**G**) mapped to it. **H** Spatial location of HSPC subpopulations showing the pseudotime in the E13.5 fetal liver sections. **I** Spatial location of HSPC subpopulations in the E13.5 fetal liver sections. **J** and **K** UMAP plots showing enrichment scores of hematopoietic stem cells and multipotent progenitor cells (HSC/MPPs) and hematopoietic progenitor cells (HPCs) signatures across different HSPCs clusters. **L** Heatmap showing gene enrichment scores of different HSPC subpopulation signatures

To investigate the more elaborate spatial distribution of distinct HSPC subpopulations, we performed further analysis of the HSPC cluster (Fig. 2A-B). Based on the expression of key feature genes, we identified 6 distinct progenitor cell populations, including HSC/MPPs, granulocyte–macrophage progenitors (GMPs), common lymphoid progenitors (CLPs), monocyte-dendritic cell progenitors (MDPs), megakaryocyte-erythroid progenitors (MEPs) and megakaryocyte progenitors (MKPs). The HSC/MPP cluster specifically expressed hallmark genes, such as *Hlf*, *Mecom*, and *Fgd5* (Fig. 2C-E). Pseudotime analysis revealed a developmental trajectory in which HSC/MPPs differentiate into GMP, CLP, and MEP populations, confirming the accurate identification of distinct HSPC subpopulations (Fig. 2F-H). Notably, all progenitor cell populations were distributed throughout the fetal liver without evidence of distinct regional localization (Fig. 2I).

Validation of these clusters was also performed by comparing our spatial transcriptomic data with a published fetal liver dataset (Li et al. 2020), which included HSC/MPPs, hematopoietic progenitor cells (HPCs), common myeloid progenitors (CMPs), GMPs, CLPs, MDPs, MEPs, MKPs, progenitor-B cells (ProB) and EryP (Fig. S2A-B). The HSC/MPP cluster exhibited the highest scores for both HSC/MPP and HPC, providing strong evidence that the HSC/MPP population was accurately identified in our spatial transcriptomic dataset (Fig. 2J-K). Similarly, the other progenitor populations also showed transcriptional signatures consistent with their corresponding clusters in the published single-cell RNA sequencing (scRNA-seq) data, further validating our clustering results (Fig. 2L). Moreover, integration of our spatial transcriptomic data with the published single-cell dataset revealed a highly coordinated organization of HSPC clusters, reinforcing the robustness of our classification (Fig. S2C-E). Notably, the HSC/MPP cluster also expressed key marker genes associated with lymphoid differentiation, including *Flt3*, *Spi1*, *Satb1*, *Ikzf1*, and *Bcl11a* (Amann-Zalcenstein et al. 2020), suggesting the presence of lymphoid-primed multipotent progenitors (LMPPs) within this population (Fig. S2F). These findings highlight the capability of SeekSpace to resolve continuous hematopoietic progenitor stages within the fetal liver.

### Resolving the spatial organization of HSC/MPPs and RPs in the fetal liver

**Fig. 3:**
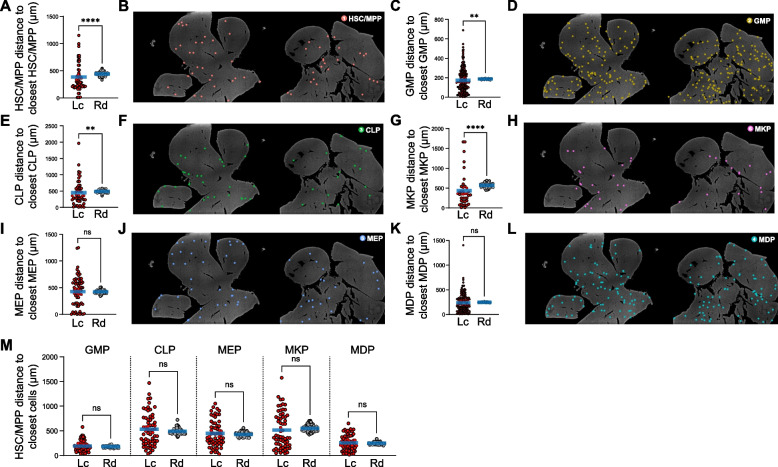
The spatial organization of HSPCs. **A** and **B** Distance analysis (**A**) and distribution (**B**) of HSC/MPPs to closest HSC/MPPs. Lc, location; Rd, randomly distributed distance. Statistical differences were calculated using two-tailed unpaired Student’s *t*-tests if the data were normally distributed or two-tailed Mann–Whitney test if they were not normally distributed (****p < 0.0001). **C** and **D** Distance analysis (**C**) and distribution (**D**) of common myeloid progenitors (CMPs) to closest CMPs. Lc, location; Rd, randomly distributed distance. Statistical differences were calculated using two-tailed unpaired Student’s *t*-tests if the data were normally distributed or two-tailed Mann–Whitney test if they were not normally distributed (**p < 0.01). **E** and **F** Distance analysis (**E**) and distribution (**F**) of common lymphoid progenitors (CLPs) to closest CLPs. Lc, location; Rd, randomly distributed distance. Statistical differences were calculated using two-tailed unpaired Student’s *t*-tests if the data were normally distributed or two-tailed Mann–Whitney test if they were not normally distributed (**p < 0.01). **G** and **H** Distance analysis (**G**) and distribution (**H**) of megakaryocyte progenitors (MKPs) to closest MKPs. Lc, location; Rd, randomly distributed distance. Statistical differences were calculated using two-tailed unpaired Student’s *t*-tests if the data were normally distributed or two-tailed Mann–Whitney test if they were not normally distributed (****p < 0.0001). **I** and **J** Distance analysis (**I**) and distribution (**J**) of megakaryocyte-erythroid progenitors (MEPs) to closest MEPs. Lc, location; Rd, randomly distributed distance. Statistical differences were calculated using two-tailed unpaired Student’s *t*-tests if the data were normally distributed or two-tailed Mann–Whitney test if they were not normally distributed (ns: not significant). **K** and **L** Distance analysis (**K**) and distribution (**L**) of monocyte-dendritic cell progenitors (MDPs) to closest MDPs. Lc, location; Rd, randomly distributed distance. Statistical differences were calculated using two-tailed unpaired Student’s *t*-tests if the data were normally distributed or two-tailed Mann–Whitney test if they were not normally distributed (ns: not significant). **M** Distance analysis of each indicated cell population to the closest HSC/MPPs. Lc, location; Rd, randomly distributed distance. Statistical differences were calculated using two-tailed unpaired Student’s *t*-tests if the data were normally distributed or two-tailed Mann–Whitney test if they were not normally distributed (ns: not significant)

To better understand the spatial organization of HSC/MPPs and restricted progenitors (RPs) in the fetal liver, we analyzed the spatial distances between different HSPC subpopulations. Our analysis revealed that not only HSC/MPPs but also several RPs, including GMPs, CLPs, and MKPs, exhibited significantly closer spatial distances to themselves compared to randomly distributed cells (Fig. 3A-H). In contrast, MEPs and MDPs showed no such spatial proximity (Fig. 3I-L). These findings suggest that both HSC/MPPs and RPs undergo active expansion in the fetal liver. This pattern starkly contrasts with the spatial distribution of HSPCs in adult bone marrow (BM), where RPs do not exhibit self-proximity, reflecting a differentiation bias (Wu et al. 2024).

We further investigated the spatial relationship between HSC/MPPs and RPs. No significant differences were observed in the spatial proximity between HSC/MPPs and RPs (GMPs, CLPs, MEPs, MKPs, and MDPs) when compared to a random distribution (Fig. 3M). This result indicates that HSC/MPPs are spatially segregated from their downstream RPs, a pattern consistent with the spatial organization of HSC/MPPs and RPs previously described in adult BM (Wu et al. 2024).

### Active expansion of HSCs in the fetal liver, but not adult BM

**Fig. 4:**
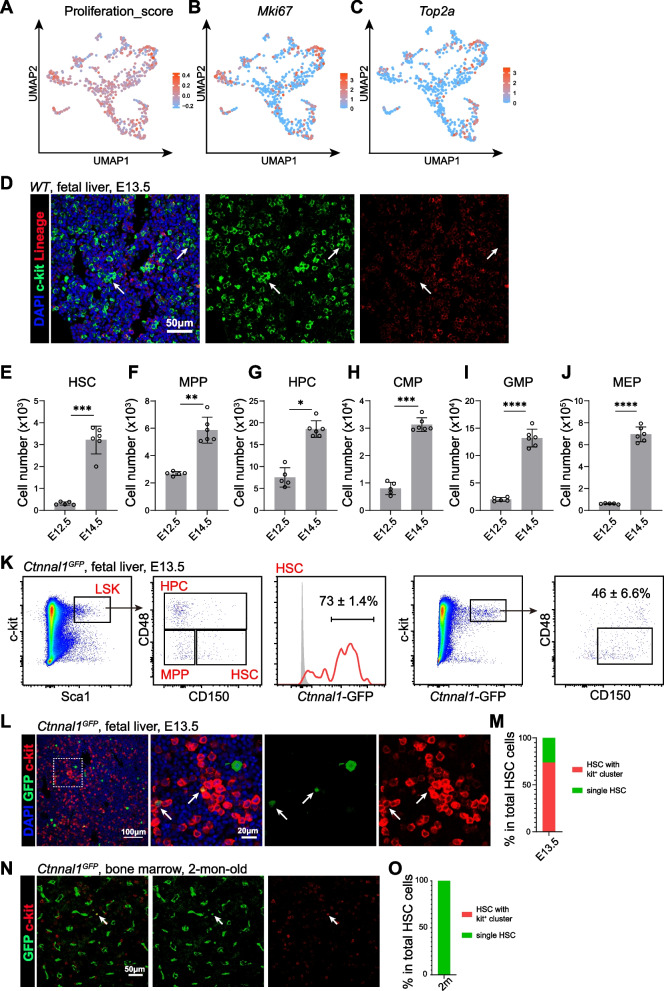
Anatomy of active expansion of HSPCs. **A**-**C** UMAP plots showing the enrichment scores of proliferation signatures and the expression of representative genes in different HSPCs clusters. **D** Confocal imaging of E13.5 fetal liver sections showing Lineage^−^c-kit^+^ hematopoietic progenitors forming cell clusters. **E**-**J** Quantification the total numbers of HSCs (**E**), MPPs (**F**), HPCs (**G**), CMPs (**H**), GMPs (**I**) and MEPs (**J**) at different developmental stages. All data represent mean ± SD from 5–8 mice per time point from 3 independent experiments (*p < 0.05, **p < 0.01, ***p < 0.001, ****p < 0.0001, ns: not significant). **K** Flow cytometric analysis of E13.5 fetal liver revealed efficient labeling of Lineage^−^c-kit^+^Sca1^+^CD48^−^CD150^+^ HSCs by GFP in *Ctnnal1*^*GFP*^ embryos. All data represent mean ± SD from 3 mice per time point from 3 independent experiments. **L** Confocal imaging of E13.5 fetal liver sections showing *Ctnnal1*-GFP^+^c-kit^+^ hematopoietic stem cells (HSCs) in proximity to c-kit^+^ hematopoietic progenitor clusters. **M** Proportions of *Ctnnal1*-GFP^+^c-kit^+^ HSCs that adjacent to c-kit^+^ hematopoietic progenitor clusters at E13.5 fetal liver sections. More than 100 HSCs were quantified across three independent experiments. **N** Confocal imaging of 2-mon-old bone marrow sections showing *Ctnnal1*-GFP^+^c-kit^+^ hematopoietic stem cells (HSCs) were observed as single cells. **O** Proportions of single *Ctnnal1*-GFP^+^c-kit^+^ HSCs at 2-mon-old bone marrow sections. More than 100 HSCs were quantified across three independent experiments

The spatial organization of cells offers insights into their functional dynamics and cell origins. Since daughter cells are typically adjacent after cell division, the distance between cells indicates their relationships (Wu et al. 2024; Zhang et al. 2021a). The spatial proximity observed among different HSPC subpopulations, including HSC/MPPs, GMPs, CLPs, and MKPs, indicates active expansion at E13.5 (Fig. 3A-H). These populations also exhibited high proliferation marker scores and expression levels of *Mki67* and *Top2a* (Fig. 4A-C and Fig. S3A-G). Consistently, numerous c-kit^+^Lineage^−^ hematopoietic progenitor cells were observed forming clusters throughout the fetal liver (Fig. 4D). To further explore these findings, we collected embryos at E12.5 and E14.5 and analyzed the cell numbers of HSCs, MPPs, HPCs, CMPs, GMPs and MEPs in the fetal liver (Fig. S3H). Between E12.5 and E14.5, the numbers of HSCs and other progenitor cell fractions increased dramatically (Fig. 4E-J). The greater number of GMPs in the fetal liver may result from both HSC/MPP differentiation and GMP proliferation (Yokomizo et al. 2022).

We next investigated the spatial distribution of HSCs during fetal hematopoiesis. A previous study demonstrated that *Ctnnal1-*GFP enriched HSCs in adult BM (Acar et al. 2015). Similarly, we found that c-kit^+^*Ctnnal1-*GFP^+^ cells corresponded to approximately half of the phenotypic CD150^+^CD48^−^Lineage^−^c-kit^+^Sca1^+^ HSCs in fetal liver at E13.5 (Fig. 4K). In situ staining of c-kit^+^GFP^+^ HSCs revealed that most HSCs were localized within clusters of c-kit⁺ progenitors (Fig. 4L-M). In contrast, in adult BM, c-kit^+^GFP^+^ HSCs were observed as isolated single cells, consistent with previous reports (Fig. 4N-O). Together, these findings suggest that HSPCs undergo significant expansion during early fetal liver hematopoiesis, a process that is not observed in adult BM.

### Distinct niche requirements for the expansion of HSC/MPPs and RPs in the fetal liver

**Fig. 5:**
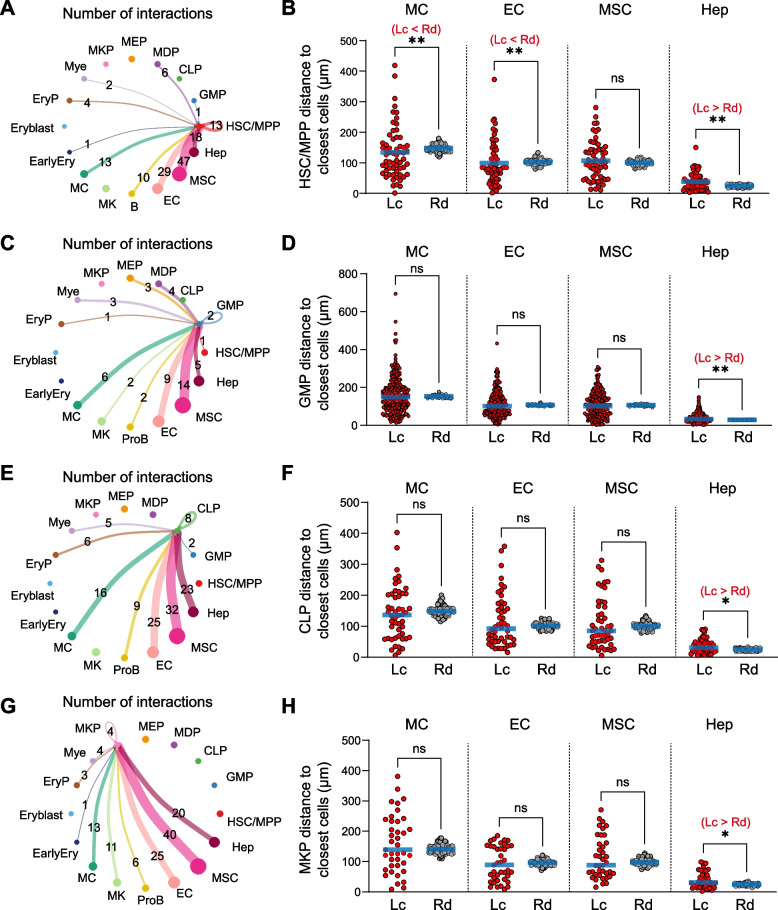
Cell–cell interactions between niche cells and HSPCs. **A** CellChat analysis showing the number of interactions between niche cells and HSC/MPPs. **B** Distance analysis of each indicated cell type to the closest HSC/MPPs. Lc, location; Rd, randomly distributed distance. Statistical differences were calculated using two-tailed unpaired Student’s *t*-tests if the data were normally distributed or two-tailed Mann–Whitney test if they were not normally distributed (**p < 0.01, ns: not significant). **C** CellChat analysis showing the number of interactions between niche cells and GMPs. **D** Distance analysis of each indicated cell type to the closest GMPs. Lc, location; Rd, randomly distributed distance. Statistical differences were calculated using two-tailed unpaired Student’s *t*-tests if the data were normally distributed or two-tailed Mann–Whitney test if they were not normally distributed (**p < 0.01, ns: not significant). **E** CellChat analysis showing the number of interactions between niche cells and CLPs. **F** Distance analysis of each indicated cell type to the closest CLPs. Lc, location; Rd, randomly distributed distance. Statistical differences were calculated using two-tailed unpaired Student’s *t*-tests if the data were normally distributed or two-tailed Mann–Whitney test if they were not normally distributed (**p < 0.01, ns: not significant). **G** CellChat analysis showing the number of interactions between niche cells and MKPs. **H** Distance analysis of each indicated cell type to the closest MKPs. Lc, location; Rd, randomly distributed distance. Statistical differences were calculated using two-tailed unpaired Student’s *t*-tests if the data were normally distributed or two-tailed Mann–Whitney test if they were not normally distributed (**p < 0.01, ns: not significant)

To comprehensively elucidate the cellular populations and signaling pathways that support fetal hematopoiesis, we employed CellChat to predict significant ligand-receptor interactions between fetal liver niche cells and different HSPC subpopulations within a 100 μm distance. Macrophages (MC), endothelial cells (EC), mesenchymal stromal cells (MSC) and hepatoblasts (Hep) exhibited the highest numbers of interactions with HSC/MPPs and their downstream RPs (Fig. 5A, C, E and G). HSC/MPPs, CLPs, and MKPs exhibited a higher number of cell–cell interactions with niche cells compared to GMPs, indicating differing levels of dependence on supporting niches. To determine whether these niche components are spatially proximal to HSPCs, we calculated the distances between various niche cell populations and different HSPC subpopulations. Our analysis revealed that MCs and ECs were significantly closer to HSC/MPPs compared to a random distribution, whereas MSCs and hepatoblasts were not (Fig. 5B). Additionally, spatial distribution patterns and colocalization scores further highlighted the physical proximity of HSC/MPPs with distinct niche components (Fig. S4). In contrast, no significant differences were observed in the spatial proximity of these niche components to downstream RPs (GMPs, CLPs, and MKPs) when compared to a random distribution (Fig. 5D, F and H). These findings suggest that HSC/MPPs, but not their downstream RPs, rely on physical proximity to their supporting niche cells.

### Spatial transcriptomic characterization of the HSPCs expansion niche

**Fig. 6:**
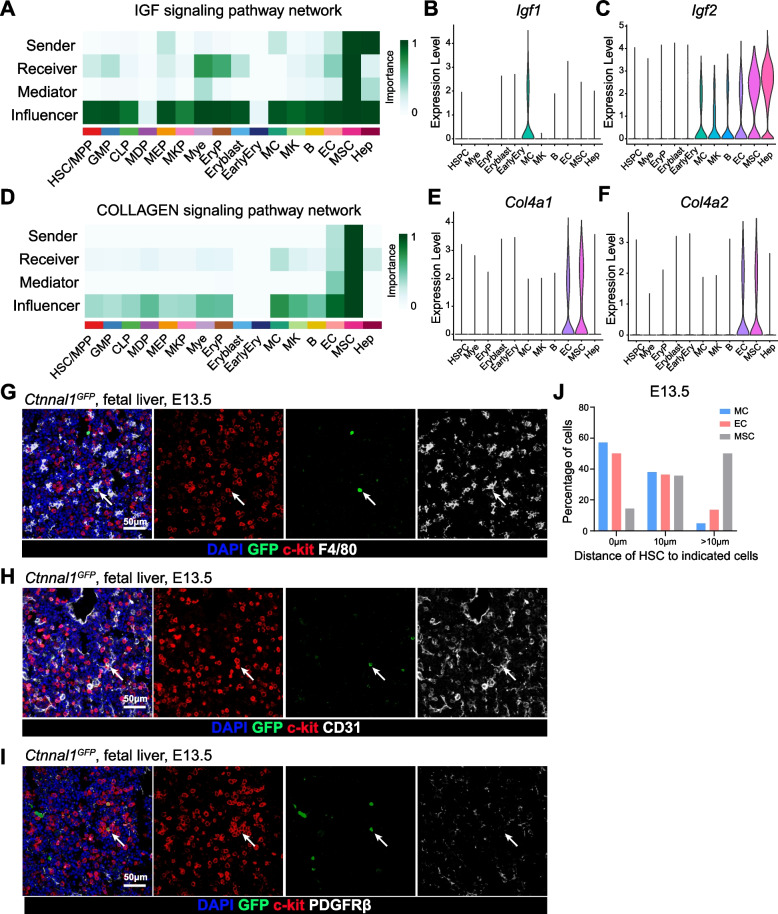
Identification of expansion niches of HSC/MPPs. **A** Heatmap showing the contribution of various cell populations to the IGF signaling pathway network. **B** and **C** Violin plots showing the expression levels of *Igf1* (B) and *Igf2* (**C**). **D** Heatmap showing the contribution of various cell populations to the COLLAGEN signaling pathway network. **E** and **F** Violin plots showing the expression levels of *Col4a1* (**E**) and *Col4a2* (**F**). **G** Confocal imaging of E13.5 fetal liver sections showing F4/80^+^ macrophages in proximity to *Ctnnal1*-GFP^+^c-kit^+^ HSCs. **H** Confocal imaging of E13.5 fetal liver sections showing CD31^+^ endothelial cells in proximity to *Ctnnal1*-GFP^+^c-kit^+^ HSCs. **I** Confocal imaging of E13.5 fetal liver sections showing PDGFRβ^+^ mesenchymal stromal cells distantly located from *Ctnnal1*-GFP^+^c-kit^+^ HSCs. **J** Distribution of distances between *Ctnnal1*-GFP^+^c-kit^+^ HSCs and F4/80^+^ macrophages, CD31^+^ endothelial cells and PDGFRβ^+^ mesenchymal stromal cells at E13.5. Over 100 HSCs were quantified in each niche component across three independent experiments

Notably, several well-established ligand-receptor interactions, previously implicated in the regulation of hematopoiesis, were identified, including *Dlk1*-*Notch1/2*, *Igf1/2*-*Igf1r* and *Col4a1/2*-*Cd44* (Fig. S5) (Burns et al. 2005; Cao et al. 2016; Mirshekar-Syahkal et al. 2013; Young et al. 2021; Zhang and Lodish 2004; Zheng et al. 2022). The interaction between MCs and HSC/MPPs was particularly enriched in the IGF signaling pathway, which is supported by the specific expression of *Igf1* and *Igf2* in MCs (Fig. 6A-C). In contrast, extracellular matrix and collagen signaling pathways were enriched in the interactions between ECs and HSC/MPPs, consistent with previous reports indicating that ECM signaling can suppress HSC differentiation (Fig. 6D-F) (Khadilkar et al. 2020; Monticelli et al. 2024). Furthermore, the spatial expression patterns of key ligand-receptor pairs, including *Igf1-Igf1r*, *Igf2-Igf1r*, *Col4a1-Cd44*, and *Col4a2-Cd44*, further supported the cell–cell interactions between HSC/MPPs and their niche components (Fig. S6). To further validate these findings, we performed in situ imaging on E13.5 fetal liver sections. Approximately half of the MCs and ECs were closely associated with c-kit^+^*Ctnnal1-*GFP^+^ HSCs, whereas MSCs were positioned at a greater distance from the HSCs (Fig. 6G-J). These observations corroborate our computational predictions and suggest that MCs and ECs play central roles in supporting HSC/MPP expansion, each through distinct mechanisms.

## Discussion

In this study, we employed a novel spatial transcriptomics framework to construct a single-cell spatial transcriptomic atlas of the fetal liver. This high-resolution dataset revealed the intricate architecture of fetal liver during fetal hematopoiesis. While previous studies have provided an anatomical overview of the fetal liver (Lu et al. 2021), the precise spatial organization of sparsely distributed HSPCs and their niche cells at true single-cell resolution has remained elusive. Here, we identify different HSPC subpopulations and characterize their spatial organization during fetal liver hematopoiesis. While SeekSpace provides distinct capabilities, its current implementation may have limitations relative to other in situ RNA capture methods. The platform could potentially miss rare cell types and be less sensitive in detecting spatially homogeneous transcriptional patterns, which may influence spatial relationship interpretations in some contexts. Integrating complementary spatial techniques could improve analytical accuracy of cellular organization in the future.

The fetal liver is a highly dynamic organ that undergoes continuous and profound changes throughout development. While previous studies have characterized fetal liver hematopoiesis at later embryonic stages (Lee et al. 2021; Li et al. 2020), the establishment of HSPC expansion niches during early fetal liver development has remained largely undefined. Our findings uncover the spatial organization of HSPCs undergoing significant clonal expansion during early fetal liver hematopoiesis. Although earlier work has documented the dramatic expansion of HSCs (Ema and Nakauchi 2000; Ganuza et al. 2022), this study is the first to define their spatial expansion organization. Notably, recent investigations have elucidated the resilient anatomical structure of hematopoiesis in adult BM (Wu et al. 2024; Zhang et al. 2021a). In contrast, our findings reveal substantial differences in the spatial organization of HSPCs between the fetal liver and adult BM, emphasizing the distinct behaviors of HSPCs in these two developmental contexts.

During development, HSCs undergo significant changes in proliferative activity, differentiation potential, transcriptomic profiles, and epigenetic landscapes (Li et al. 2020). Beyond intrinsic regulatory mechanisms, temporal shifts in extrinsic niche cues play a pivotal role in shaping HSC fate decisions (Guo et al. 2025; Lee et al. 2022; Mesquita Peixoto et al. 2025). The observed correlation between changes in HSC niches and fate specification highlights the intricate coordination between intrinsic HSPC dynamics and extrinsic niche remodeling during fetal liver development. By integrating ST analysis and in situ imaging, we found that HSC/MPPs and their downstream RPs have differing levels of dependence on supporting niches. These findings provide a foundation for advancing finely tuned ex vivo HSC culture systems.

## Materials and methods

### Mouse strains

All mice used in this study were maintained on a C57BL/6 background at the Animal Facility of the Center for Excellence in Molecular Cell Science (CEMCS), Chinese Academy of Sciences. The *Ctnnal*^*GFP*^ (Acar et al. 2015) mouse strains have been previously described. Embryos were generated by timed matings and staged according to embryonic day. All mice were housed in a specific pathogen-free animal facility under a 12-h light/12-h dark cycle, and all experimental protocols were approved by the Institutional Animal Care and Use Committees of CEMCS.

### Flow cytometry

Liver hematopoietic cells were isolated by crushing fetal liver between two glass slides, followed by passage through 25-G syringes containing Ca^2+^ and Mg^2+^ free HBSS supplemented with 2% heat-inactivated fetal bovine serum to obtain a single-cell suspension. The cell suspension was then filtered through a 40-μm nylon mesh. Freshly prepared cells were incubated with antibodies in 100 μl ice-cold HBSS (Ca^2+^ and Mg^2+^ free) containing 2% heat-inactivated fetal bovine serum (FACS buffer). The following antibodies were used for staining: anti-CD150 (TC15 - 12 F12.2), anti-CD48 (HM48 - 1), anti-Sca1 (E13 - 161.7), anti-c-kit (2B8), anti-CD34 (SA376 A4), anti-CD16/32 (93) and lineage antibody cocktail (anti-Ter119 (TER- 119), anti-B220 (6B2), anti-Gr- 1 (8 C5), anti-CD2 (RM2 - 5), anti-CD3 (17 A2), anti-CD5 (53–7.3), and anti-CD8 (53–6.7)). Hematopoietic stem cells (HSCs) were defined as lineage^−^Sca1^+^c-kit^+^CD48^−^CD150^+^. Multipotent progenitor cells (MPPs) were defined as lineage^−^Sca1^+^c-kit^+^CD48^−^CD150^−^. Hematopoietic progenitor cells (HPCs) were defined as lineage^−^Sca1^+^c-kit^+^CD48^+^. DAPI was used to exclude dead cells. Antibodies were from eBioscience or BioLegend. Antibody staining was performed for 30 min on ice. Flow cytometric analysis was performed on LSRFortessa (BD Biosciences) or CytoFlex LX (Beckman Coulter). Data were analyzed using FlowJo software. All data represent mean ± SD from more than 3 embryos from three independent experiments.

### Immunostaining and confocal imaging

Freshly dissected tissues were fixed in 4% paraformaldehyde (PFA, Solarbio, p1110) overnight at 4 °C. For antigens sensitive to prolonged fixation, a 3–4 h incubation was used. Tissues were cryoprotected by overnight infiltration with 30% sucrose, embedded in optimal cutting temperature (OCT, Thermo scientific, NEG- 50–6502), and stored at − 80 °C. Cryosections of 10 mm in thickness were collected on positively charged slides and stored at − 80 °C until use. Sections were blocked in PBS containing 5% donkey serum and 0.1% Triton X- 100 for 1 h, followed by overnight incubation at 4 °C with primary antibodies. After washing with PBS, sections were incubated with secondary antibodies and DAPI (Sigma, D9542, 1:1000) for 4 h at 4 °C. Antibodies used included: anti-CD31 (R&D, AF3628, 1:250), anti-PDGFRβ (eBioscience, 14–1402 - 82, 1:100), anti-F4/80 (R&D, MAB5580, 1:200), anti-c-kit (R&D, AF1356, 1:200), anti-GFP (Aves Labs, GFP- 1020, 1:400), donkey anti-chicken Alexa Fluor 488 (Jackson ImmunoResearch, 703–545 - 155, 1:500), donkey anti-rat Alexa Fluor 647 (Jackson ImmunoResearch, 712–605 - 153, 1:500), donkey anti-rabbit Alexa Fluor 647 (Invitrogen, A31573, 1:500), donkey anti-goat Alexa Fluor 488 (Invitrogen, A11055, 1:500) and donkey anti-goat Alexa Fluor 647 (Invitrogen, A21447, 1:500). Non-immune immunoglobulins of the same isotype as the primary antibodies were used as negative controls. Finally, slides were mounted with ProLong™ Gold anti-fade reagent (Invitrogen, P36930), and images were acquired on a Leica TCS SP8 WLL or Leica TCS SP8 STED confocal microscope. The distances between *Ctnnal1*-GFP^+^c-kit^+^ candidate hematopoietic stem cells (HSCs) and F4/80^+^ macrophages, PDGFRβ^+^ mesenchymal stromal cells, or CD31^+^ endothelial cells were measured using ImageJ software. The number of *Ctnnal1*-GFP^+^c-kit^+^ candidate HSCs in proximity to c-kit^+^ hematopoietic progenitor clusters was counted based on whether direct-contact existed. More than 100 HSCs were quantified from three independent experiments at each time point.

### Cell nuclei with spatial barcodes preparation

Single-cell nuclei suspension with spatial barcodes was prepared from fresh frozen tissues using the SeekSpace® Single Cell Spatial Transcriptome-seq Kit (K02501 - 08). Briefly, tissues were cryo-sectioned to 10–20 μm on a cryostat (Leica) at − 20 °C. The regions of interest were placed on the SeekSpace® Chip, ensuring that no tissue folds occurred. A finger was placed on the back of the SeekSpace® Chip to melt the tissue. The SeekSpace® Chip was placed in the SeekSpace® sc-Spatial Chip Holder and incubated at 37 °C for 90 s. Following this, a Space Chamber was placed on the chip, and 150 µl of labeling reagent was added without introducing bubbles. Next, the tissue sections were fixed, fluorescence photographed and homogenized in pre-chilled lysis buffer using a Dounce homogenizer (KIMBLE #D8938). After washing and filtration, the number of nuclei was estimated using a Fluorescence Cell Analyzer (Seekgene #M002B) with AO/PI reagent, and the suspension was kept on ice until further use.

### Sequencing library preparation and sequencing

Single-nucleus RNA-seq library and spatial barcode library were prepared using the SeekSpace® Single Cell Spatial Transcriptome-seq Kit (K02501 - 08) according to the manufacturer's instructions. Briefly, nuclei were evenly divided into 8 PCR tubes, and reverse transcription was performed on 600–30,000 nuclei per tube using different reverse transcription primers. Fifteen cycles of annealing (ramping from 8 °C to 42 °C) were performed to enhance primer hybridization and intracellular reverse transcription efficiency. After reverse transcription, the nuclei were washed twice to remove residual primers and pooled together. Subsequently, an appropriate number of nuclei were combined with ligation reagents and added to the sample wells of the SeekOne® DD Chip S3 (Chip S3). Barcoded Hydrogel Beads (BHBs) and partitioning oil were dispensed into the corresponding wells. The cell-containing ligation reagents and BHBs were encapsulated into emulsion droplets using the SeekOne® Digital Droplet System. Immediately after transferring the emulsion droplets into PCR tubes, the droplets were incubated at 20 °C for 60 min followed by 10-min heat inactivation at 65 °C to obtain barcoded cDNA and spatial barcodes. The barcoded cDNA and spatial barcodes were then decrosslinked and recovered from the droplets. To obtain more template-switched cDNA, a second round of reverse transcription was performed followed by a PCR pre-amplification. The pre-amplified product was used for both spatial barcode library construction and cDNA library construction. Finally, sample indexes were added to the pre-amplified product during spatial barcode library construction via PCR. After cDNA purification, 20 ng of cDNA was amplified by index PCR. The indexed sequencing libraries were cleaned using VAHTS DNA Clean Beads (Vazyme N411) and analyzed using Qubit (Thermo Fisher Scientific Q33226) and a Bio-Fragment Analyzer (Bioptic, Qsep400). The single- nucleus RNA-seq library and spatial barcode library were sequenced on an Illumina NovaSeq 6000 with PE150 read length.

### Single-cell spatial transcriptomic data analysis

Aligned reads and gene-barcode matrices were generated from FASTQ files (Read 1, Read 2 and i7 index) using Cell Ranger software package (version 3.0) with default parameters. Further analysis and visualization were performed using R package Seurat (version 4.1.1) (Hao et al. 2021). Quality control was performed to filter low-quality cells. Cells with more than 100 and less than 3000 genes and had < 1% of their transcripts mapped to mitochondrial genes were retained for downstream analysis. The LogNormalize method was applied to normalize expression of each cell. Then, top principal components were selected using the elbow method and utilized for dimensionality reduction and unsupervised clustering. t-Distributed Stochastic Neighbor Embedding (tSNE) and uniform manifold approximation and projection (UMAP) were used for visualization. Differentially expressed genes (DEGs) across clusters were identified using the FindAllMarkers function in Seurat, applying a one-sided Wilcoxon rank-sum test with Bonferroni correction for multiple testing. Genes with an avg_logFC (log fold-change of the average expression) > 0.5 and adjusted *P* < 0.05 were considered significant. Nebulosa package (version 0.99.92) was used to visualize the gene expression based on kernel density estimation. To compare transcriptomic signatures of HSPC subpopulations from spatial transcriptomic data and published single-cell RNA sequencing data, the top 100 DEGs (log_2_ fold-change) of each HSPC population were used. Gene enrichment scores were calculated using Seurat’s AddModuleScore function, and the average gene enrichment scores were calculated and visualized using heatmaps. CCA-based integration method in Seurat was used to integrated spatial transcriptomic dataset and published single-cell dataset. The genes for the proliferation score were sourced from a previously reported list of common proliferation markers (*Zwint*, *E2f1*, *Fen1*, *Foxm1*, *H2afv*, *Hmgb2*, *Mcm2*, *Mcm3*, *Mcm4*, *Mcm5*, *Mcm6*, *Mki67*, *Mybl2*, *Pcna*, *Plk1*, *Ccnd1*, *Aurka*, *Bub1*, *Top2a*, *Tyms*, *Dek*, *CcnB1* and *CcnE1*) (Whitfield et al. 2006). Cellular interaction analysis was performed using CellChat v2 (version 2.1.0) (Jin et al. 2023). Compared to the original CellChat, the updated CellChat v2, enables inference of physically proximal cell–cell communication between interacting cell groups when spatial locations of cells are available. To find cell–cell interactions that have closer physical distance, we restricted the cell–cell interactions within a 100 μm distance.

### Spatial organization analysis

To better visualize the spatial organization of individual nucleus, we map nuclei onto DAPI imaging according to their spatial locations with plotly package (4.10.3). This method was adapted with DimPlot and FeaturePlot functions in Seurat. Euclidean distances between cells were calculated based on their spatial locations. Random distributions of cells were simulated by randomly selecting dots corresponding to hematopoietic cell types at the frequencies observed in the spatial transcriptomic data. Distances between these random cells and the indicated cells were measured, and each random simulation was repeated 100 times. RANN package (version 2.6.1) was used to calculate the nearest neighbours for every cell. Cauchy kernel function was used to calculate the colocalization score.

### Quantification and statistical analysis

Data presented in the figures represent results from multiple independent experiments conducted on different days using different mice. Results are presented as means ± SD and statistical analyses were performed using GraphPad Prism version 8.2.1. For graphs showing distances between cells in fetal liver section, we indicate the mean and each dot corresponds to one cell. Statistical analyses between two samples were performed by using Student’s *t* test if the data were normally distributed and Mann–Whitney test if the data were not normally distributed. To compare differences among multiple groups, statistical analysis was determined by one-way ANOVA with Tukey’s multiple comparisons tests.

## Supplementary Information

Supplementary Material 1. Fig. S1 Detected gene number of different cell populations in fetal liver. (A and B) Violin plots showing the number of detected genes (A) and unique molecular identifiers (UMIs; B) in different clusters. Fig. S2 Validation of distinct HSPC subtypes with scRNA-sequencing. (A) UMAP visualization of unsupervised clustering of HSPCs from scRNA-seq dataset. (B) Dot plot showing the expression levels of representative marker genes in different HSPCs clusters. (C and D) UMAP visualization of unsupervised clustering of integrated spatial transcriptomics (ST) data and scRNA-seq data. (E) Dot plot showing the expression levels of representative marker genes across different HSPC clusters. (F) UMAP plots displaying the expression of representative genes of LMPPs in different HSPCs clusters. Fig. S3 The proliferation signatures of HSPCs in fetal liver. (A-C) UMAP plots showing the enrichment scores of proliferation signatures and the expression of representative genes in different HSPCs clusters from published scRNA-seq data. (D-F) UMAP plots showing the enrichment scores of proliferation signatures and the expression of representative genes in different HSPCs clusters within the integrated dataset. (G) Spatial location of HSPC subpopulations showing the proliferative scores on the tissue sections. (H) Flow cytometric analysis of E12.5 fetal liver showed the gating strategies of HSC, MPP, HPC, CMP, GMP and MEP. Fig. S4 The spatial location of HSC/MPPs and their niche components in fetal liver. (A-D) Spatial mapping of HSC/MPPs and various niche components, highlighting their proximity within tissue sections. (E-I) Spatial distribution of different cell populations, displaying colocalization scores with HSC/MPPs. Fig. S5 The fetal liver niche for HSPCs expansion. (A-D) Dot plot showing significant putative ligand/receptor pairs between niche cells and HSC/MPPs (A), GMPs (B), CLPs (C) and MKPs (D). Fig. S6 The expression patterns of critical ligand-receptor pairs. (A-D) Spatial location of different cell population showing the expression patterns of indicated ligand-receptor pairs on the tissue section.

## Data Availability

The raw single-cell spatial transcriptomic (scST) data have been deposited in Genome Sequence Archive (GSA; https://ngdc.cncb.ac.cn/gsa/) under accession number CRA021089 and are publicly available as of the date of publication. Reviewers can access the raw data via the following link: https://ngdc.cncb.ac.cn/gsa/s/p31FsnWD. The processed scST data have been deposited in the OMIX database under accession number OMIX008192, and reviewers can access these data through the following link: https://ngdc.cncb.ac.cn/omix/preview/NDk34H3K.

## Abbreviations

HSPCs: Hematopoietic stem and progenitor cells
ECs: Endothelial cells
EHT: Endothelial-to-hematopoietic transition
HCCs: Hematopoietic cluster cells
ST: Spatial transcriptomics
HSC/MPPs: Hematopoietic stem cells and multipotent progenitor cells
RPs: Restricted progenitors
snRNA-seq: Single-nucleus RNA sequencing
UMIs: Unique molecular identifiers
UMAP: Uniform manifold approximation and projection
Mye: Myeloid cells
EryP: Erythroid progenitors
Eryblast: Erythroblasts
EarlyEry: Early erythrocytes
MC: Macrophages
MK: Megakaryocytes
MSC: Mesenchymal stromal cells
Hep: Hepatoblasts
GMPs: Granulocyte-macrophage progenitors
CLPs: Common lymphoid progenitors
MDPs: Monocyte-dendritic cell progenitors
MEPs: Megakaryocyte-erythroid progenitors
MKPs: Megakaryocyte progenitors
HPCs: Hematopoietic progenitor cells
CMPs: Common myeloid progenitors
ProB: Progenitor-B cells
BM: Bone marrow
LMPPs: lymphoid-primed multipotent progenitors
scRNA-seq: Single-cell RNA sequencing
HSCs: Hematopoietic stem cells
EMPs: Erythro-myeloid progenitors
