# Supplementary figures and images for: Resolving the spatial organization of fetal liver hematopoiesis by SeekSpace
Source: Cell Regen. 2025 Apr 22;14:15. doi: 10.1186/s13619-025-00234-0 (PMC12014969; doi:10.1186/s13619-025-00234-0)

**A**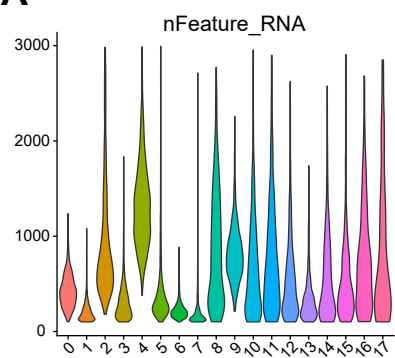**B**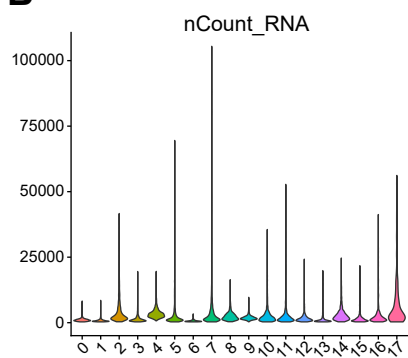**Fig. S1**

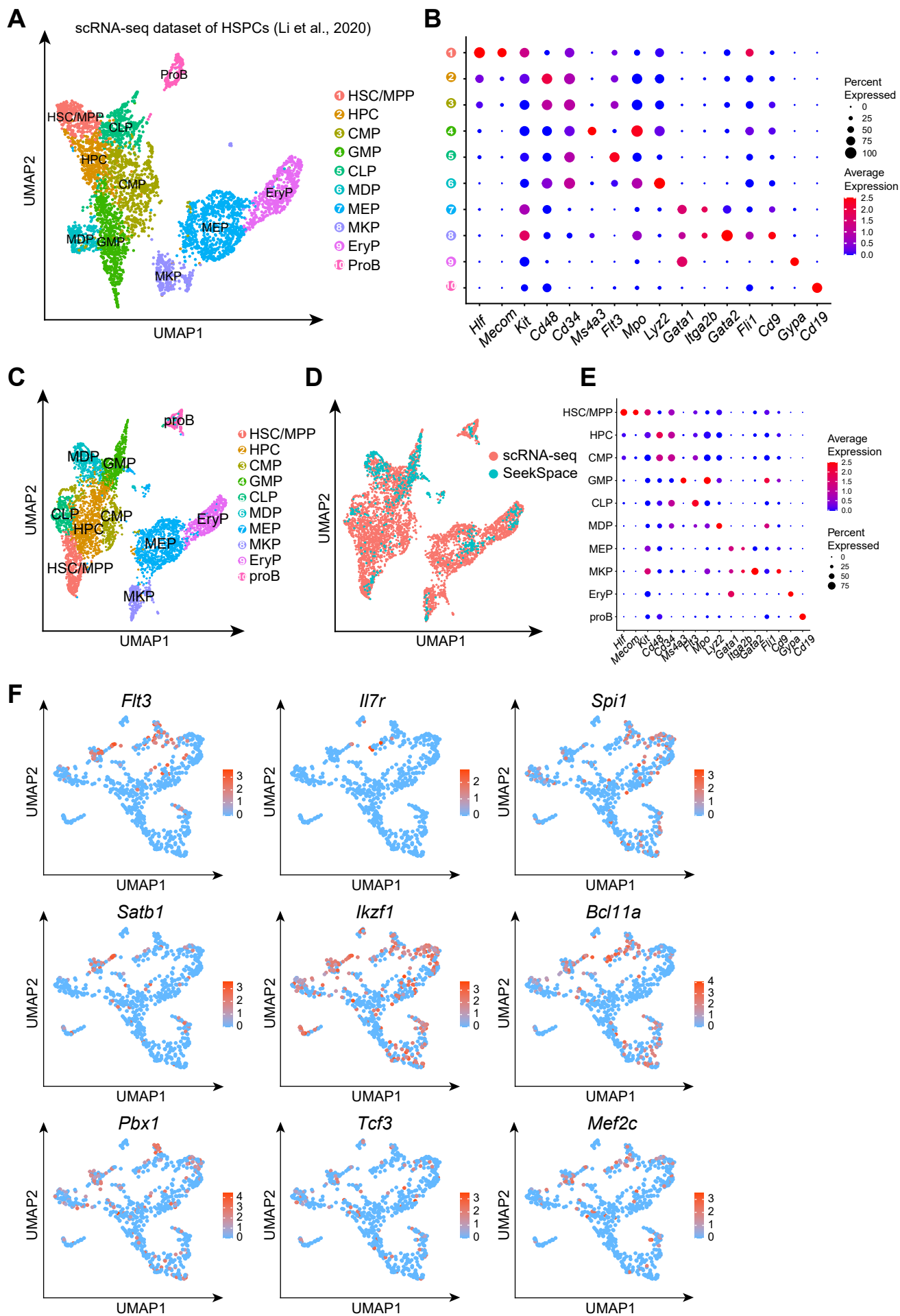

**Fig. S2**

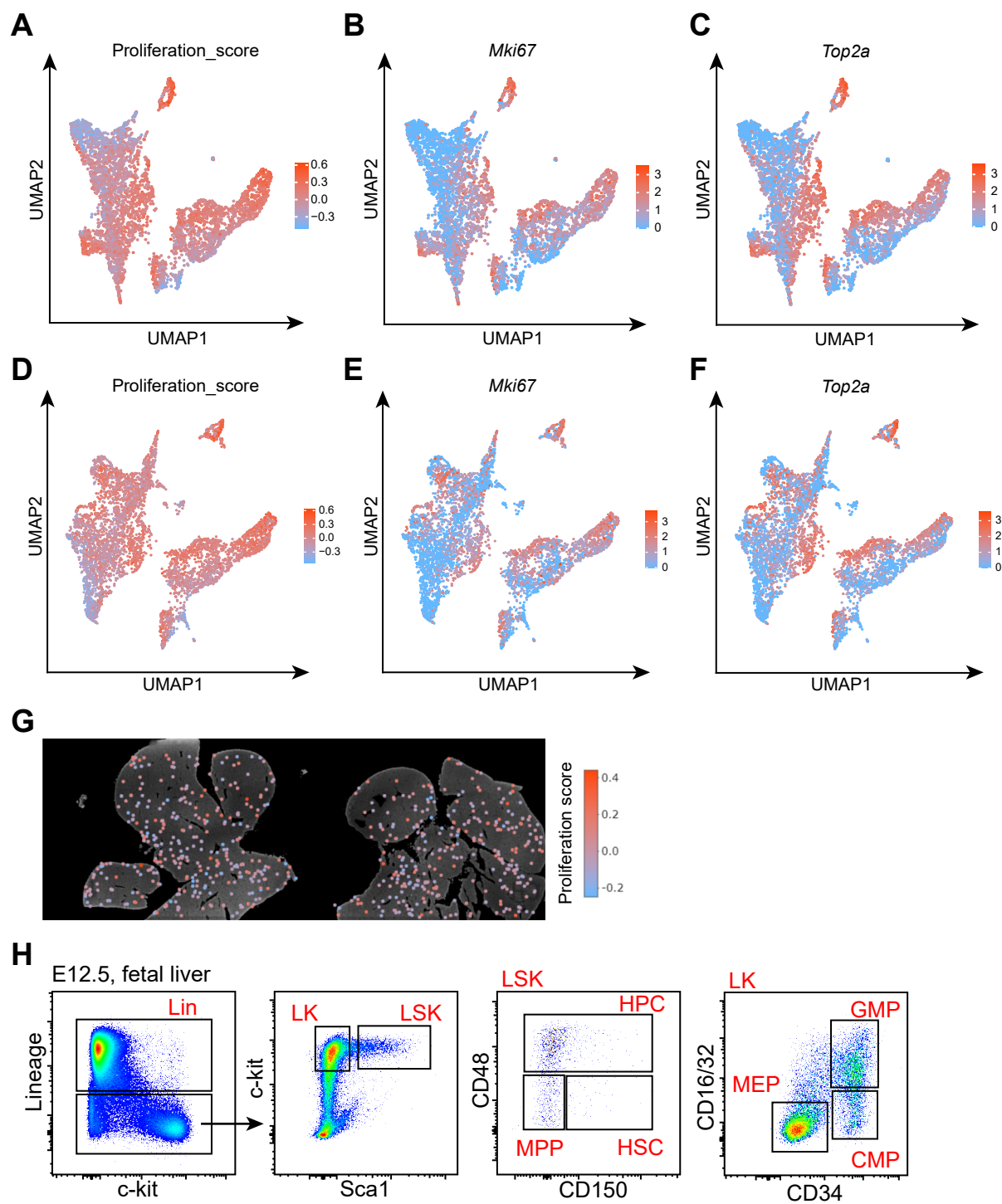

**Fig. S3**

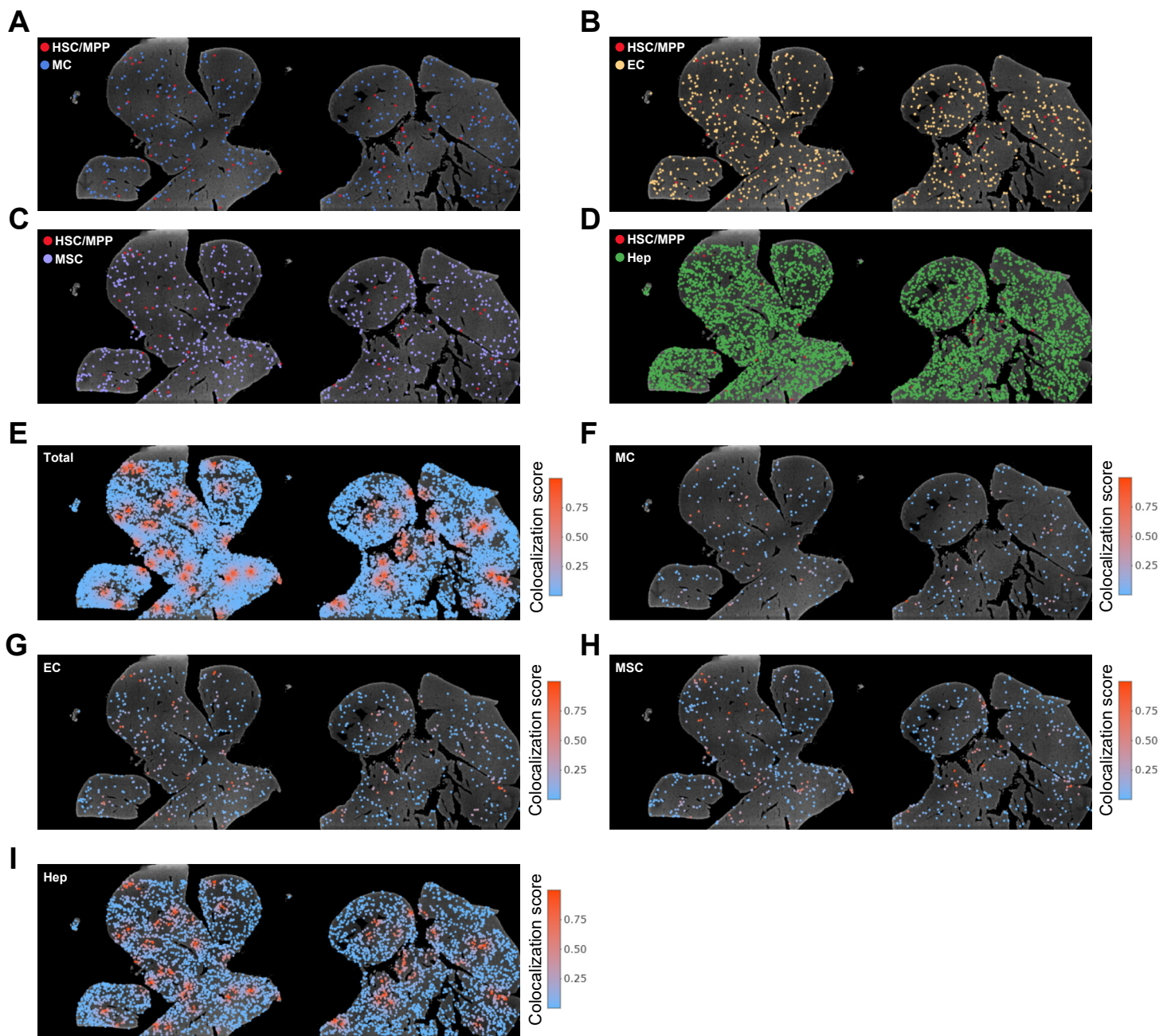

**Fig. S4**



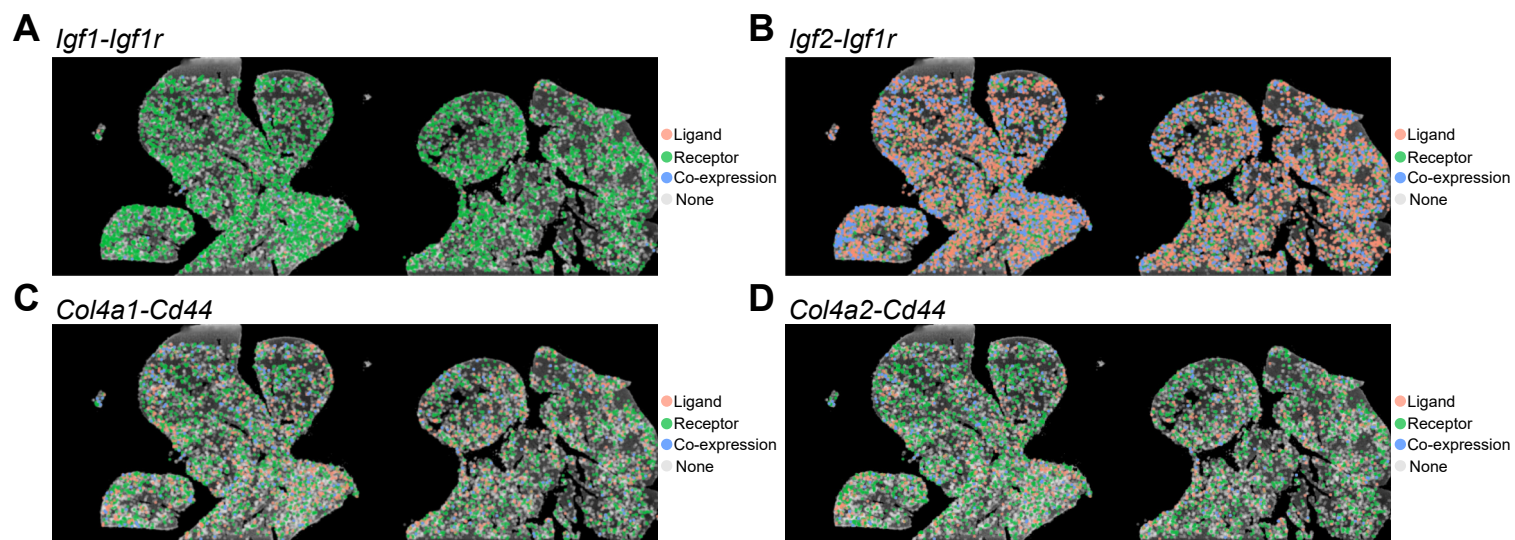

**Fig. S6**

Supplement: Supplementary file 1 — Supplementary Material 1. Fig. S1 Detected gene number of different cell populations in fetal liver. (A and B) Violin plots showing the number of detected genes (A) and unique molecular identifiers (UMIs; B) in different clusters. Fig. S2 Validation of distinct HSPC subtypes with scRNA-sequencing. (A) UMAP visualization of unsupervised clustering of HSPCs from scRNA-seq dataset. (B) Dot plot showing the expression levels of representative marker genes in different HSPCs clusters. (C and D) UMAP visualization of unsupervised clustering of integrated spatial transcriptomics (ST) data and scRNA-seq data. (E) Dot plot showing the expression levels of representative marker genes across different HSPC clusters. (F) UMAP plots displaying the expression of representative genes of LMPPs in different HSPCs clusters. Fig. S3 The proliferation signatures of HSPCs in fetal liver. (A-C) UMAP plots showing the enrichment scores of proliferation signatures and the expression of representative genes in different HSPCs clusters from published scRNA-seq data. (D-F) UMAP plots showing the enrichment scores of proliferation signatures and the expression of representative genes in different HSPCs clusters within the integrated dataset. (G) Spatial location of HSPC subpopulations showing the proliferative scores on the tissue sections. (H) Flow cytometric analysis of E12.5 fetal liver showed the gating strategies of HSC, MPP, HPC, CMP, GMP and MEP. Fig. S4 The spatial location of HSC/MPPs and their niche components in fetal liver. (A-D) Spatial mapping of HSC/MPPs and various niche components, highlighting their proximity within tissue sections. (E-I) Spatial distribution of different cell populations, displaying colocalization scores with HSC/MPPs. Fig. S5 The fetal liver niche for HSPCs expansion. (A-D) Dot plot showing significant putative ligand/receptor pairs between niche cells and HSC/MPPs (A), GMPs (B), CLPs (C) and MKPs (D). Fig. S6 The expression patterns of critical ligand-rece [file 13619_2025_234_MOESM1_ESM.pdf]
